# Mitochondrial dysfunction in diabetic cardiomyopathy: a review of pathogenic mechanisms and therapeutic strategies

**DOI:** 10.3389/fcvm.2026.1751243

**Published:** 2026-03-12

**Authors:** Yulian Huang, Qiao Ma, Wei Wang, Shanjun Huang, Jianqiang Zhao

**Affiliations:** 1 Department of Nursing, The Fourth Affiliated Hospital of School of Medicine, and International School of Medicine, International Institutes of Medicine, Zhejiang University, Yiwu, China; 2 Department of Cardiology, The Fourth Affiliated Hospital of School of Medicine, and International School of Medicine, International Institutes of Medicine, Zhejiang University, Yiwu, China

**Keywords:** cell death, diabetic cardiomyopathy, metabolic remodeling, mitochondrial dysfunction, oxidative stress, targeted interventions

## Abstract

Diabetic cardiomyopathy (DCM) presents a significant clinical challenge, independently contributing to heart failure morbidity and mortality in patients with diabetes mellitus. Although advancements in glycemic control and cardiovascular therapies have been made, effective strategies specifically addressing DCM remain limited, highlighting the urgent need to clarify its underlying pathogenesis. Recent research has increasingly recognized mitochondrial dysfunction as a central driver of DCM, linking metabolic derangements, oxidative stress, inflammation, and programmed cell death into a complex pathological network. This review critically examines recent experimental and clinical findings to delineate the multidimensional mechanisms by which mitochondrial impairment propels DCM progression. We specifically explore alterations in energy metabolism, excessive reactive oxygen species (ROS) production, inflammasome activation, and dysregulation of apoptotic and ferroptotic pathways. Additionally, we summarize the latest advances in mitochondria-targeted therapeutic strategies, including small molecule antioxidants, metabolic modulators, gene-based therapies, stem cell-derived exosomes, and lifestyle interventions aimed at restoring mitochondrial health. Finally, we briefly highlight future research directions, emphasizing the potential of multi-targeted interventions and emerging technologies such as single-cell transcriptomics to deepen mechanistic insights. A comprehensive understanding of mitochondrial-centered pathways may offer promising avenues for innovative therapies and improved clinical outcomes in DCM.

## 1 Introduction

Diabetic cardiomyopathy (DCM) is characterized by structural and functional abnormalities of the myocardium that arise directly from diabetes mellitus, independent of overt coronary artery disease, hypertension, or valvular heart disease (1). A comprehensive analysis reported that the global prevalence of diabetes in adults rose from 7% in 1990 to 14% in 2022, with projections indicating a continued upward trend (2). Epidemiological studies highlight DCM as a major contributor to heart failure and cardiovascular mortality in diabetic individuals, frequently progressing from asymptomatic diastolic dysfunction to overt systolic impairment (3–5). Despite intensified glycemic control and advancements in cardiovascular therapeutics, no specific intervention has been shown to effectively prevent or reverse DCM, underscoring the need for a deeper understanding of its pathophysiological underpinnings.

Traditionally, hyperglycemia-induced metabolic derangements, endothelial dysfunction, and microvascular damage have been implicated in DCM development (6). However, clinical studies have revealed that strict glycemic control alone is insufficient to prevent the onset and progression of DCM; myocardial alterations can persist even in patients who maintain well-regulated glycated hemoglobin levels (5). This has prompted a paradigm shift toward exploring alternative pathological drivers that operate across multiple biological dimensions.

In recent years, mitochondrial dysfunction has emerged as a central, unifying mechanism in DCM pathogenesis. Beyond their role in adenosine triphosphate (ATP) production, cardiac mitochondria are critical for regulating redox balance, calcium signaling, apoptosis, and lipid metabolism (7–10). Under diabetic conditions, cardiomyocytes exhibit disrupted mitochondrial bioenergetics, excessive reactive oxygen species (ROS) generation, impaired mitochondrial dynamics, and defective mitophagy, forming a pathological network that accelerates myocardial injury (11–13).

Although several studies and reviews have addressed individual aspects of mitochondrial involvement in DCM, a comprehensive and integrative overview that links pathogenic mechanisms with translational therapeutic strategies remains lacking. Furthermore, the advent of omics technologies and mitochondria-targeted therapies necessitates an updated synthesis of the evolving landscape.

This review aims to fill this gap by providing a multidimensional analysis of mitochondrial dysfunction in DCM, encompassing its roles in metabolic remodeling, oxidative stress, inflammation, and cell death. We also highlight emerging mitochondria-targeted interventions—including small-molecule antioxidants, gene-based therapies, stem cell-derived exosomes, and lifestyle modifications—along with prospective applications of single-cell and spatial transcriptomics for mechanistic discovery and precision therapy development. By bridging molecular insights with clinical relevance, this review offers a conceptual framework to guide future research and therapeutic innovation in DCM.

## 2 Pathological features of DCM

The clinical and pathological manifestations of DCM are multifaceted, encompassing structural, metabolic, and functional derangements. Critically, emerging evidence positions mitochondrial dysfunction as a central upstream driver that orchestrates these diverse pathological features. The following sections detail these hallmark changes, with an emphasis on their root connection to mitochondrial impairment, thereby setting the stage for a deeper mechanistic discussion.

### 2.1 Structural alterations of the myocardium

Myocardial fibrosis represents one of the earliest and most prominent pathological features of DCM (14). Animal studies using streptozotocin (STZ)-induced diabetic rat models have shown a significant increase in the expression of type I and type III collagen in cardiac tissue compared to controls (15), with Masson's trichrome staining revealing extensive collagen deposition in the interstitial regions and a concomitant decline in myocardial compliance (16). Clinical investigations have similarly demonstrated that the myocardial interstitial collagen volume index (CVI) in diabetic patients is elevated and correlates positively with the severity of diastolic dysfunction (17).

In addition to extracellular matrix remodeling, diabetic cardiomyopathy also involves intrinsic cardiomyocyte alterations, which is another significant histological manifestation of DCM (18). Experimental studies reported a roughly 25% increase in the cross-sectional area of left ventricular cardiomyocytes in diabetic rats, accompanied by ventricular wall thickening (19, 20). Imaging studies further indicated that nearly 45% of patients with type 2 diabetes exhibit ventricular hypertrophy, suggesting compensatory structural remodeling (21).

Nevertheless, cardiomyocyte apoptotic burden is markedly increased. Terminal deoxynucleotidyl transferase dUTP nick end labeling (TUNEL) staining analysis revealed that the apoptotic index in diabetic rat myocardium is significantly higher than that of controls, along with elevated Bax expression, reduced Bcl-2 levels, and enhanced cytochrome c release (22). Clinical biopsy findings have also confirmed a significantly higher proportion of TUNEL-positive cardiomyocytes in diabetic patients with heart failure compared to non-diabetic counterparts (23).

Collectively, these histopathological alterations—fibrosis, hypertrophy, and increased apoptosis—not only contribute to myocardial remodeling and functional impairment in DCM, but also serve as potential therapeutic targets for disease modification. Critically, these structural changes are increasingly understood as consequences of sustained mitochondrial distress, including energy crises, excessive ROS production, and the activation of mitochondrial-dependent cell death pathways.

### 2.2 Metabolic alterations in the myocardium

Alterations in myocardial metabolic patterns constitute a hallmark feature of DCM. Studies have shown that plasma free fatty acid (FFA) levels in patients with type 2 diabetes are significantly elevated compared to healthy controls, promoting a preferential reliance on fatty acid oxidation for cardiac energy supply (24, 25). Consistently, experimental studies in STZ-induced diabetic rat models have reported a significant increase in myocardial fatty acid oxidation rates, accompanied by upregulation of metabolic genes such as CPT-1 and PPARα (26, 27). Although fatty acid oxidation can partially sustain ATP production, it is less efficient, requires higher oxygen consumption, and leads to the accumulation of lipid intermediates, thereby exacerbating lipotoxic injury in cardiomyocytes (28). This preferential shift towards fatty acid oxidation demands greater oxygen consumption per unit ATP produced, further burdening the diabetic heart with compromised oxygen delivery.

Meanwhile, the capacity for glucose oxidation is markedly impaired (29). Clinical evidence indicates that myocardial glucose uptake in diabetic patients is reduced significantly, accompanied by concomitant decreases in the activities of key glycolytic and oxidative enzymes, such as pyruvate dehydrogenase (PDH) (30). Integrated metabolomic analyses have further revealed that intermediate metabolites of the tricarboxylic acid (TCA) cycle, including citrate and succinate, are decreased by 10%–20% in diabetic animal hearts, reflecting widespread disruption of aerobic energy metabolism (31).

This substrate shift, characterized by enhanced fatty acid oxidation and suppressed glucose utilization, not only compromises cardiac energy efficiency and reduces metabolic flexibility but also directly reflects and exacerbates mitochondrial metabolic inflexibility, providing a critical therapeutic target for DCM progression (32, 33).

### 2.3 Cardiac functional impairments

In the early stages of DCM, diastolic dysfunction predominates (34). Studies have demonstrated that even with preserved left ventricular ejection fraction (LVEF), patients with diabetes exhibit an approximately 20%–30% elevation in left ventricular end-diastolic pressure (LVEDP), reflecting reduced cardiac compliance and impaired early ventricular filling (35). As the disease progresses, a subset of patients develops systolic dysfunction. Clinical data indicate that around 15%–20% of individuals with type 2 diabetes experience a decline in LVEF to below 55%, accompanied by decreased cardiac output, suggesting the onset of left ventricular systolic impairment (36). Correspondingly, in STZ-induced diabetic rat models, a reduction in LVEF by approximately 10%–15% has been observed, alongside an increase in left ventricular end-diastolic dimension (LVEDd) and marked ventricular wall thickening (37).

These functional abnormalities not only compromise exercise tolerance but are also strongly associated with higher rates of heart failure and cardiovascular mortality. The progression from diastolic to systolic dysfunction can be traced, in part, to the cumulative burden of mitochondrial failure-compromised ATP synthesis for contraction, ROS-mediated damage to contractile proteins, and calcium mishandling—linking the cellular energetic deficit to the organ-level functional decline (32).

In summary, DCM is characterized by a triad of pronounced abnormalities: structural remodeling (fibrosis, hypertrophy, apoptosis); metabolic inflexibility (fuel shift toward fatty acids); and functional decline (diastolic and systolic dysfunction). Crucially, these pathological features converge on mitochondrial dysfunction, which will be discussed in detail in the following section.

## 3 Mechanistic dissection: mitochondrial dysfunction as a hierarchical network

While mitochondrial dysfunction manifests across multiple dimensions in DCM, these abnormalities are not equivalent in their origin or timing. We propose a hierarchical framework to delineate primary drivers from secondary or compensatory adaptations. At the foundation lie the primary insults: the diabetic milieu of chronic hyperglycemia and lipotoxicity directly instigates mitochondrial metabolic dysfunction (fuel shift and impaired efficiency) and exacerbated oxidative stress (excessive ROS production), constituting the core initial injury (24, 38).

In response to this injury, adaptive quality control mechanisms are engaged, primarily involving mitochondrial dynamics and mitophagy. These processes aim to segregate damage, remove dysfunctional organelles, and maintain a healthy network. However, under unrelenting metabolic stress, these initially compensatory responses become maladaptive and dysfunctional-leading to excessive fission and defective mitophagy-thus transitioning into secondary pathologies that amplify the initial injury (39, 40).

**Figure 1:**
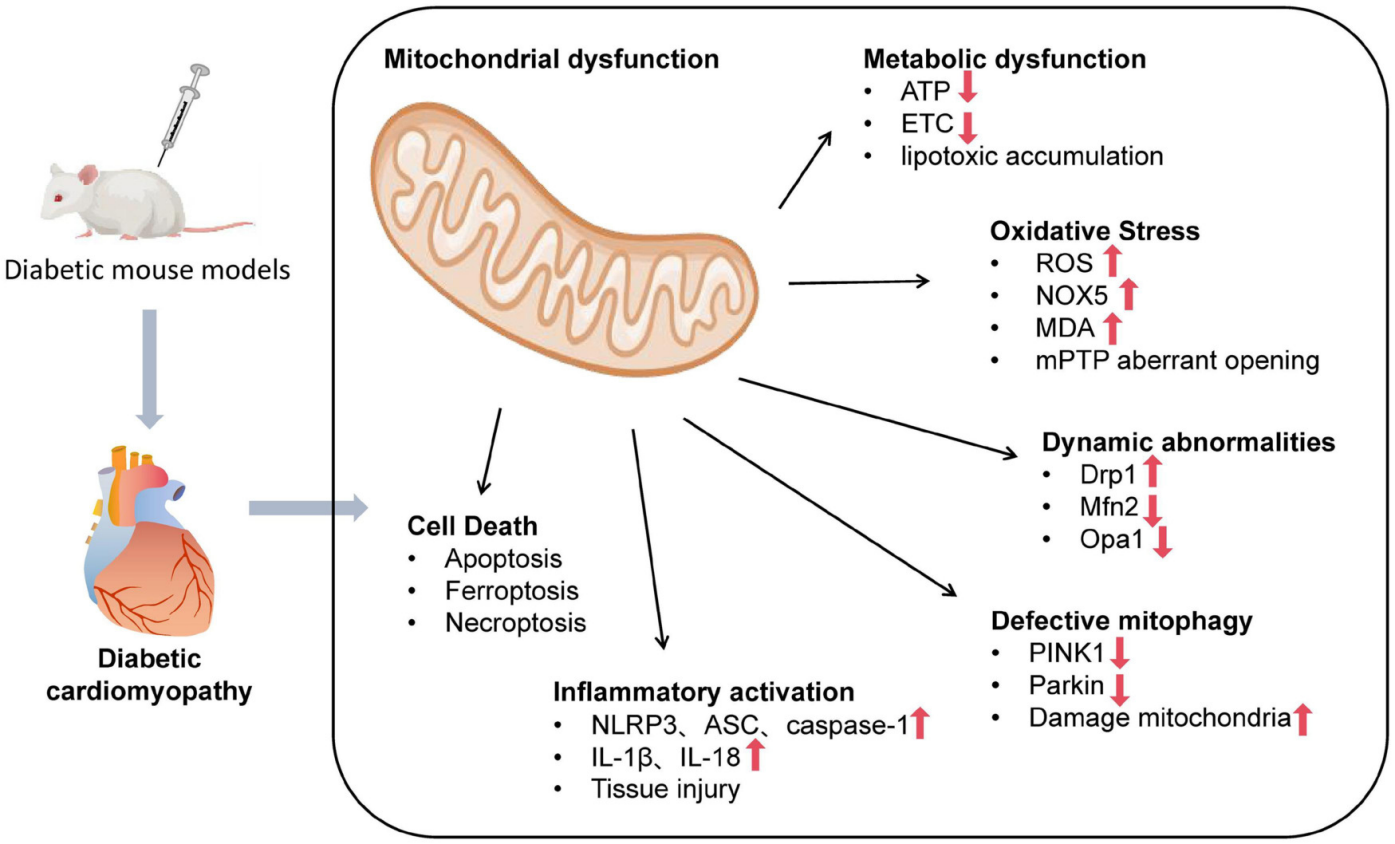
Schematic overview of mitochondrial dysfunction-centered pathogenic network in diabetic cardiomyopathy.

This cascade of primary and secondary mitochondrial failures collectively precipitates downstream cellular catastrophes, including the activation of multiple programmed cell death pathways. This hierarchical framework is summarized in Figure 1, which provides a graphical overview of the multi-layered network linking primary metabolic insults to terminal cell death events in DCM. The following sections detail each layer of this hierarchical network, emphasizing their interconnections.

### 3.1 Primary layer: metabolic dysfunction and oxidative stress

#### 3.1.1 Mitochondrial energy metabolic dysfunction

Given the critical dependence of cardiomyocytes on mitochondrial energy production, mitochondrial metabolic dysfunction emerges as a pivotal early feature in the pathogenesis of DCM (38). Studies in high-fat diet (HFD)-induced diabetic mouse models have shown that myocardial ATP levels decrease by approximately 25% compared to controls, accompanied by significant inhibition of electron transport chain (ETC) complex III activity (41). Impaired ATP synthesis directly compromises myocardial contractile efficiency, with early increases in left ventricular end-diastolic pressure (LVEDP), underscoring a close link between metabolic disturbances and cardiac dysfunction (42).

Concurrently, myocardial substrate utilization undergoes substantial metabolic reprogramming (43). Clinical metabolic imaging studies have revealed that in diabetic patients, myocardial fatty acid uptake increases by approximately 30%–40%, while glucose uptake declines by more than 40%, as assessed by positron emission tomography (PET) imaging (44). This preferential reliance on fatty acid oxidation, although partially sustaining ATP production, leads to elevated oxygen consumption and reduced energetic efficiency, exacerbating mitochondrial burden and metabolic stress (38).

Further investigations indicate significant accumulation of fatty acid metabolism intermediates such as acylcarnitine and diacylglycerol in the myocardium of HFD-fed mice (45). This lipotoxic accumulation induces mitochondrial membrane hyperpolarization and dysfunction of inner membrane proteins, establishing a vicious cycle of energy production impairment and oxidative stress. Notably, these changes can be detected at early stages of DCM, highlighting mitochondrial metabolic imbalance as a critical initiating event in disease progression.

#### 3.1.2 Oxidative stress and ROS accumulation

Mitochondria-derived ROS accumulation plays a pivotal role in the progression of DCM. Under diabetic conditions, sustained hyperglycemia and lipid metabolic disturbances impair the efficiency of the electron transport chain (ETC), leading to increased electron leakage (38). Studies in db/db mouse models have demonstrated that mitochondrial ROS levels in the myocardium are elevated by approximately twofold compared to controls, accompanied by a marked reduction in complex I activity (46), suggesting that metabolic stress and electron transfer defects synergistically drive oxidative stress.

Excessive ROS not only directly damages mitochondrial membranes but also activates redox-sensitive signaling pathways, thereby exacerbating cellular dysfunction. Analysis of myocardial tissue from diabetic patients revealed that the expression of NADPH oxidase 5 (NOX5), a NADPH oxidase isoform expressed in humans but absent in rodents, is markedly increased (47), further amplifying ROS production and mitochondrial stress. Moreover, levels of malondialdehyde (MDA), a marker of lipid peroxidation, are significantly elevated by about 1.5–2.0-fold in diabetic myocardial tissue (48), indicating aggravated oxidative injury to membrane lipids.

Importantly, ROS accumulation has been shown to trigger aberrant opening of the mitochondrial permeability transition pore (mPTP), resulting in loss of mitochondrial membrane potential and disruption of intracellular calcium homeostasis, a phenomenon repeatedly observed in myocardial tissues from diabetic patients (49). Mitochondrial membrane dysfunction not only impairs ATP synthesis but also triggers apoptotic cascades, suggesting that targeting mPTP stabilization could represent a potential therapeutic strategy to attenuate cardiomyocyte injury in DCM (50).

### 3.2 Secondary layer: failure of quality control

#### 3.2.1 Mitochondrial dynamic abnormalities

The dynamic balance between mitochondrial fission and fusion is essential for maintaining cardiomyocyte energy metabolism and structural integrity (39). Under diabetic conditions, this balance is disrupted. Studies have demonstrated that the expression of the mitochondrial fission-related protein Dynamin-related protein 1 (Drp1) is increased in cardiac tissue, accompanied by pronounced mitochondrial fragmentation (51). Drp1 recruitment to the mitochondrial outer membrane is enhanced, facilitating mitochondrial fission through Guanosine Triphosphatease (GTPase)-driven membrane constriction. Excessive mitochondrial fission results in smaller, more numerous mitochondria, leading to reduced ATP production and increased ROS generation (52).

Concurrently, mitochondrial fusion is impaired. In diabetic hearts, the expression levels of fusion proteins such as Mitofusin 2 (Mfn2) and Optic Atrophy 1 (Opa1) are significantly decreased (53). Impaired fusion restricts the exchange of mitochondrial contents and hampers the repair of mitochondrial damage, thereby reducing cellular resilience to metabolic stress. Ultrastructural analysis further corroborates that mitochondrial fragmentation and disorganization observed in diabetic models are closely associated with cardiomyocyte functional deterioration (54).

#### 3.2.2 Impairment of mitophagy

Mitophagy, the selective autophagic removal of damaged mitochondria, is crucial for maintaining mitochondrial quality and ensuring metabolic homeostasis in cardiomyocytes (40). Studies using type 2 diabetic rat models have demonstrated that the expression levels of PTEN induced putative kinase 1 (PINK1) and Parkin in cardiac tissue are reduced by approximately 30%–40% compared to controls, correlating with the accumulation of dysfunctional mitochondria (55).

Under physiological conditions, PINK1 accumulates on the outer membrane of depolarized mitochondria, recruiting Parkin to mediate ubiquitination and facilitate autophagosome recognition and degradation of damaged organelles (56). However, under diabetic stress, reduced PINK1 stability and impaired Parkin recruitment hinder the timely clearance of defective mitochondria (57). Electron microscopy analysis revealed a substantial presence of swollen mitochondria with disrupted cristae structures in diabetic cardiomyocytes, indicative of blocked autophagic flux and mitochondrial debris accumulation (58).

The accumulation of malfunctioning mitochondria perpetuates ROS production and proinflammatory signaling, exacerbates intracellular metabolic stress, and triggers energy crises and cell death pathways. These processes collectively accelerate myocardial structural remodeling and functional decline, providing a pathological basis for the progression of DCM (50).

### 3.3 Terminal layer: activation of mitochondrial-dependent cell death

In DCM, mitochondrial dysfunction promotes multiple forms of programmed cardiomyocyte death, thereby accelerating cardiac functional deterioration (59).

First, mitochondrial-dependent apoptosis has been widely observed in human cardiomyocyte H9c2 cells cultured under high-glucose conditions. Studies have shown that after 48 h of high-glucose stimulation, cytochrome c release is markedly increased, and caspase-3 activity is significantly elevated (60), indicating that changes in mitochondrial membrane permeability initiate classical apoptotic signaling. Excessive apoptosis results in a significant loss of cardiomyocytes, compromising overall cardiac contractile performance.

Second, mitochondria are central executors of ferroptosis, a lipid peroxidation-dependent form of cell death that is markedly amplified in the diabetic myocardium (61). Mitochondrial dysfunction fuels ferroptosis through multiple interconnected routes. As the primary source of cellular ROS, dysfunctional mitochondria directly supply the initiating radicals that attack polyunsaturated fatty acids (PUFAs) in membrane phospholipids (62). Concurrently, the diabetic metabolic shift enriches the mitochondrial pool of long-chain PUFAs, such as arachidonic acid and adrenic acid, which serve as preferred substrates for lipid peroxidation (63). Concurrently, impaired mitochondrial antioxidant systems, particularly the Glutathione-glutathione peroxidase 4 (GSH-GPX4) axis, fail to effectively eliminate lipid hydroperoxides, allowing peroxidative reactions to propagate catastrophically (64, 65).

Critically, mitochondrial iron accumulation acts as a potent catalyst for this process. This mitochondrial iron overload has two major consequences. First, it directly catalyzes the Fenton reaction, converting mitochondrial H_2_O_2_ into highly toxic •OH that launches lipid peroxidation *in situ*. Second, it disrupts the function of iron-sulfur cluster (ISC) proteins in the electron transport chain, further exacerbating ROS leakage and energy deficit (66, 67). Thus, the mitochondrion becomes a nexus where excess iron and metabolic stress converge to promote ferroptotic damage.

Ferroptosis does not occur in isolation but engages in extensive cross-talk with other mitochondrial-driven death pathways in DCM, creating a synergistic network of cardiomyocyte loss. With apoptosis, lipid peroxidation products like 4-hydroxynonenal (4-HNE) can directly modify and inhibit anti-apoptotic Bcl-2 proteins, sensitizing cells to mitochondrial outer membrane permeabilization (MOMP) and cytochrome c release (68). With necroptosis, massive membrane rupture during ferroptosis releases damage-associated molecular patterns (DAMPs) that can amplify inflammatory signaling and potentiate RIPK3/MLKL-mediated necroptosis in neighboring cells (69). This death pathway synergy means that inhibiting a single pathway may be insufficient; the interplay suggests a need for combination therapeutic strategies targeting shared upstream drivers, such as mitochondrial ROS and iron overload.

The interplay of these death pathways constitutes a critical pathological basis for the transition from cellular injury to irreversible myocardial remodeling in DCM.

### 3.4 Multiscale interactions: beyond the mitochondrion

Mitochondrial abnormalities interact with other organelles and systemic pathways, amplifying injury.

#### 3.4.1 Endoplasmic reticulum stress and mitochondria-associated crosstalk

Under physiological conditions, the endoplasmic reticulum (ER) and mitochondria maintain close physical and functional interactions through mitochondria-associated membranes (MAMs), which are crucial for regulating lipid metabolism, calcium (Ca^2+^) signaling, and cellular energy homeostasis (70). The stability of mitochondria-associated membranes (MAMs) plays a vital role in preserving the metabolic balance of cardiomyocytes.

In the diabetic state, both the structure and function of MAMs become compromised. In the hearts of ob/ob mice, a genetic model of obesity-induced diabetes, MAM integrity is disrupted, leading to impaired Ca^2+^ signaling between the endoplasmic reticulum and mitochondria (71). Disruption of MAMs results in cytosolic Ca^2+^ accumulation and mitochondrial Ca^2+^ overload, which destabilizes mitochondrial membrane potential, enhances ROS production, and exacerbates energy metabolic disturbances (72).

Meanwhile, the imbalance of calcium homeostasis within the ER triggers activation of the unfolded protein response (UPR), inducing a state of chronic ER stress. Analysis of myocardial tissue has revealed that UPR-related molecules, such as GRP78 and CHOP, are significantly upregulated in diabetic models (73), indicating persistent ER stress. This bidirectional crosstalk between ER stress and mitochondrial dysfunction forms a pathological feedback loop that promotes cardiomyocyte injury and metabolic derangement (74).

Collectively, MAMs disruption acts as a critical nexus in DCM, linking and amplifying mitochondrial injury and ER stress responses, thereby driving further deterioration of cardiac function.

#### 3.4.2 Inflammatory activation

Mitochondrial dysfunction serves as a major trigger for inflammatory activation in cardiomyocytes by promoting oxidative stress (75). Studies have demonstrated that in the myocardium of Akita mice, a genetic model of insulin-deficient diabetes, the expression levels of NOD-like receptor protein 3 (NLRP3) inflammasome components NLRP3, apoptosis-associated speck-like protein containing a CARD (ASC) and caspase-1 are significantly elevated compared to controls, accompanied by increased secretion of proinflammatory cytokines such as Interleukin-1β (IL-1β) and Interleukin-18 (IL-18) (76), indicating a close link between mitochondrial dysfunction and local inflammatory responses.

Accumulation of mitochondrial ROS is recognized as a key upstream signal for NLRP3 inflammasome activation (77). The initial oxidative stress facilitates inflammasome assembly, and the subsequent release of IL-1β and IL-18 further stimulates ROS-generating systems, including NADPH oxidase 2 (NOX2), forming a ROS–inflammation–ROS positive feedback loop (78).

This vicious cycle exacerbates mitochondrial membrane potential loss, impairs ATP production, and promotes cardiomyocyte necrosis. Persistent local inflammation and metabolic imbalance ultimately lead to structural remodeling and functional deterioration of the heart, laying a pathological foundation for the progression of DCM.

#### 3.4.3 Inter-organ metabolic signaling

Emerging evidence suggests that the pathogenesis of DCM involves not only local metabolic disturbances within the heart but also systemic regulation through inter-organ metabolic signaling pathways (79). Clinical and experimental studies have demonstrated that hepatic dysregulation leads to increased secretion of lipid metabolites such as ceramides, which circulate to the myocardium and induce mitochondrial dysfunction and lipid peroxidation (80). This “liver–heart axis” highlights the pivotal role of hepatic metabolic disturbances in DCM progression.

Additionally, adipose tissue, functioning as an endocrine organ, secretes adipokines such as leptin and adiponectin, whose expression becomes dysregulated under diabetic conditions (81). Clinical studies have reported that plasma adiponectin levels are significantly reduced in diabetic patients (82), correlating with impaired mitochondrial biogenesis and disrupted energy metabolism in the myocardium. These findings underscore the critical contribution of the “adipose–heart axis” to myocardial metabolic reprogramming.

This cross-organ metabolic regulation emphasizes the systemic nature of DCM and suggests that future therapeutic strategies should move beyond localized interventions. Comprehensive modulation of the metabolic networks connecting the liver, adipose tissue, and heart may be essential to restore metabolic homeostasis and effectively halt or reverse disease progression.

## 4 Therapeutic strategies: from mitochondrial targeting to systemic modulation

From a mechanistic perspective, current therapeutic strategies for DCM can be broadly categorized into interventions that directly target mitochondrial structure and function, and those that indirectly modulate mitochondrial health by correcting upstream metabolic or signaling abnormalities.

### 4.1 Direct mitochondrial-targeted interventions

Direct mitochondrial-targeted interventions aim to preserve mitochondrial integrity, redox balance, and quality control by acting within or in close proximity to mitochondria.

Oxidative stress plays a central role in the pathogenesis of DCM, making antioxidant strategies targeting mitochondrial overproduction of ROS a critical area of early therapeutic exploration (83). Small-molecule antioxidants such as Mitoquinol Mesylate (MitoQ) and Visomitin (SkQ1) are capable of selectively accumulating within mitochondria, where they scavenge ROS and mitigate oxidative damage to lipids, proteins, and mitochondrial DNA (mtDNA) (84). Beyond preclinical models, clinical studies have also indicated that mitochondrial-targeted antioxidants such as MitoQ may reduce systemic oxidative stress and improve cardiovascular parameters in humans (85), highlighting their translational potential in DCM therapy.

In addition, intracellular levels of nicotinamide adenine dinucleotide (NAD^+^), a key electron carrier, are markedly depleted under diabetic conditions. Supplementation with NAD^+^ precursors, such as nicotinamide mononucleotide (NMN) or nicotinamide riboside (NR), can effectively replenish intracellular NAD^+^ pools, activate mitochondrial deacetylases such as silent mating type information regulation2 homolog-3 (SIRT3), and enhance energy metabolism and antioxidant defenses (86, 87).

In recent years, gene therapy and RNA-based interventions have shown significant promise in modulating mitochondrial function in DCM. Small RNA molecules, such as microRNAs (miRNAs) and small interfering RNAs (siRNAs), can target and regulate genes associated with mitochondrial dynamics (e.g., Drp1, Mfn2) or apoptotic pathways (e.g., Bcl-2 family proteins), thereby improving mitochondrial morphology and functionality (88). Experimental studies have further demonstrated that administration of a miR-195 inhibitor in diabetic rat models downregulates Drp1 expression, reduces mitochondrial fragmentation, alleviates myocardial oxidative stress, and significantly enhances left ventricular function (89).

Moreover, the application of Clustered Regularly Interspaced Short Palindromic Repeats associated protein 9 (CRISPR-Cas9) gene-editing technology presents a promising avenue for the precise correction of mitochondrial-associated gene dysfunctions. In preclinical studies involving animal models and cellular systems, CRISPR-Cas9-mediated editing of mitochondrial regulatory genes, such as MFN2, has been employed to explore the modulation of mitochondrial antioxidant defenses and energy metabolism (90). Although the current use of CRISPR technology remains predominantly restricted to experimental settings, it offers substantial potential for the accurate rectification of mitochondrial pathological alterations and for halting the progression of DCM (91).

### 4.2 Indirect metabolic and signaling modulation of mitochondrial function

Indirect strategies improve mitochondrial function by modulating systemic metabolism, intracellular signaling pathways, or intercellular communication that secondarily influence mitochondrial homeostasis.

Metabolic modulation represents a vital strategy for preserving mitochondrial function in DCM. Activation of AMP-activated protein kinase (AMPK), for example by agents such as metformin, enhances glucose uptake and optimizes fatty acid oxidation, thereby improving myocardial energy utilization efficiency (92). *In vitro* studies have also demonstrated that metformin upregulates AMPK activity and mitigates mitochondrial dysfunction and apoptosis in high glucose-treated cardiomyocytes (93). Moreover, AMPK activation promotes mitochondrial autophagy (mitophagy) and biogenesis, contributing to the maintenance of mitochondrial quality and delaying metabolic derangements in cardiomyocytes (92).

Activation of SIRT3, a mitochondrial deacetylase, offers another promising avenue for therapeutic intervention. SIRT3 modulates the deacetylation of key metabolic enzymes and antioxidant proteins, such as superoxide dismutase 2 (SOD2), thereby reinforcing mitochondrial oxidative defense systems (94). Consistently, experimental studies have shown that combined activation of Sirtuin 1 (SIRT1) and SIRT3 reduces myocardial oxidative stress and improves mitochondrial function and cardiac performance in diabetic rat models (95). As a central regulator of mitochondrial homeostasis and cellular metabolic adaptation, SIRT3 activation is regarded as a promising future target for DCM therapy.

Mesenchymal stem cell (MSC)-derived conditioned medium (CM) has been investigated for its mitoprotective effects in cardiac injury models due to its enrichment in bioactive factors and immunomodulatory properties. Studies have shown that administration of MSC-CM significantly upregulates the expression of peroxisome proliferator-activated receptor gamma coactivator 1-alpha (PGC-1α) in myocardial tissue, promotes mitochondrial biogenesis, and enhances ATP content (96), thereby improving myocardial energy metabolism and mitigating oxidative stress. Although these findings were obtained in myocardial reperfusion injury models, they highlight the therapeutic potential of MSC-derived factors in preserving mitochondrial function under cardiac pathological conditions.

In recent years, stem cell-derived exosomes, small extracellular vesicles secreted by cells, have emerged as novel mediators of intercellular communication. These exosomes are rich in miRNAs, proteins, and mitochondrial regulatory factors and can be internalized by cardiomyocytes via endocytosis (97). Animal studies have demonstrated that exosomes derived from human umbilical cord mesenchymal stem cells (HUCMSC-Exos) can activate the AMPK-ULK1 signaling pathway in diabetic myocardium, enhance autophagic activity, inhibit cardiomyocyte apoptosis, and improve myocardial energy metabolism (98). Compared with direct stem cell transplantation, exosome therapy exhibits lower immunogenicity and greater controllability. Overall, stem cells and their derived exosomes provide a promising intervention strategy for repairing mitochondrial dysfunction in DCM and have become a major focus of current preclinical research.

Aerobic exercise has been extensively validated as an effective approach to enhance both the quantity and functionality of mitochondria within cardiomyocytes. Studies have shown that eight weeks of moderate-intensity aerobic exercise training significantly increases myocardial PGC-1α expression levels in diabetic rats (99), thereby promoting mitochondrial biogenesis, improving myocardial resilience, and enhancing energy metabolism efficiency. Exercise exerts these benefits partly through the activation of AMPK and SIRT1 signaling pathways, collectively improving mitochondrial quality and antioxidant capacity, and thereby delaying myocardial functional decline.

In addition, specific dietary interventions, such as ketogenic diets and intermittent fasting, have demonstrated potential in ameliorating mitochondrial dysfunction in the diabetic heart. Animal studies have shown that ketogenic diets can balance mitochondrial dynamics, inhibit cardiomyocyte apoptosis, reduce oxidative stress, and improve myocardial energy metabolism (100, 101). Intermittent fasting, through periodic energy stress, activates the AMPK/SIRT1 signaling axis, promotes mitophagy and mitochondrial renewal, and thereby mitigates lipotoxicity and oxidative stress in diabetic cardiomyocytes (102).

**Figure 2:**
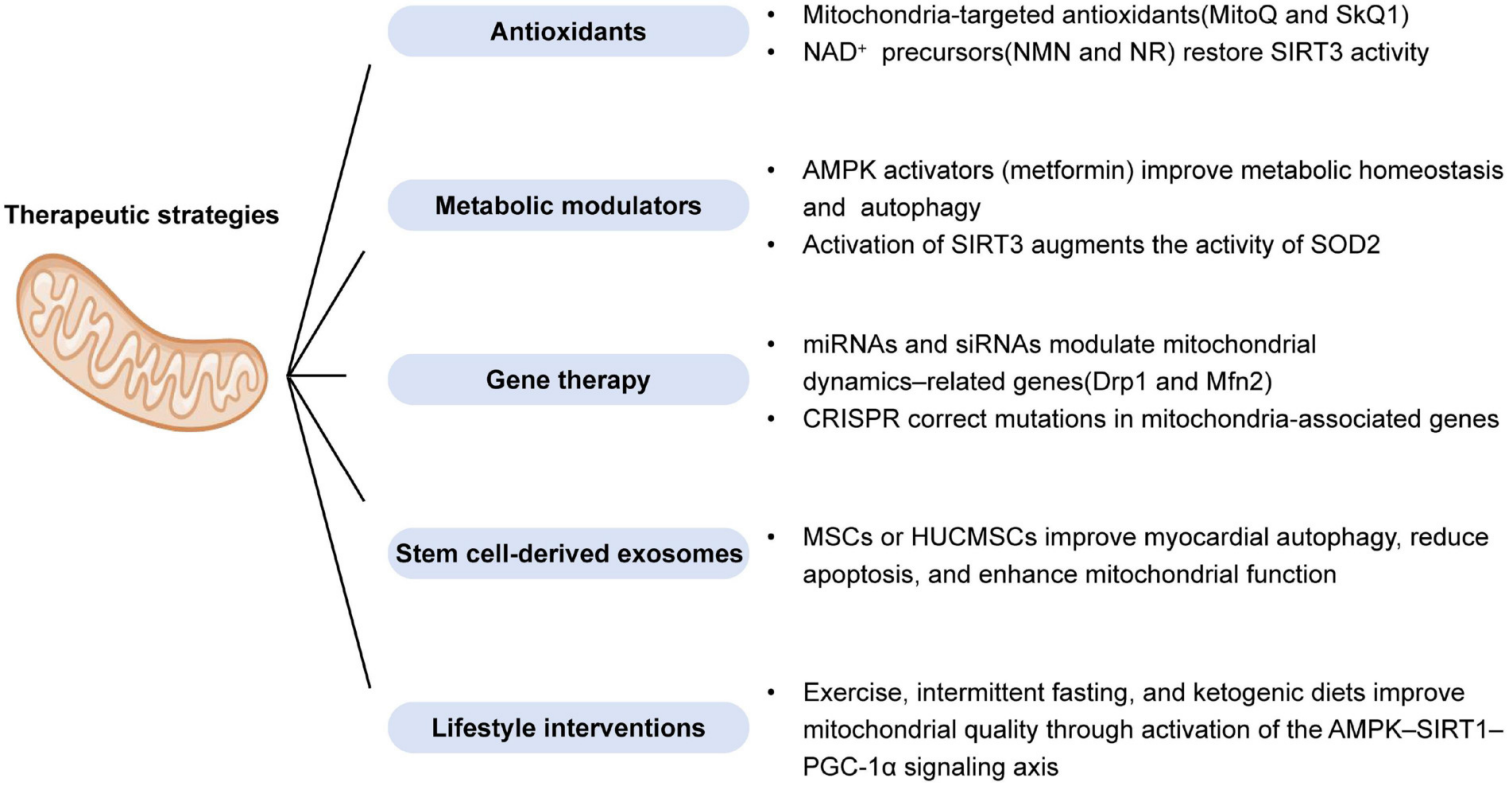
Overview of mitochondrial-targeted and mitochondria-modulating therapeutic strategies in diabetic cardiomyopathy.

Thus, lifestyle optimization strategies based on exercise and dietary interventions provide low-cost, highly feasible adjunctive therapeutic options for patients with DCM and hold significant potential for clinical application. However, clinical translation remains challenging due to limited and inconsistent trial efficacy, constraints in bioavailability and cardiac mitochondrial delivery, and substantial patient heterogeneity. Figure 2 provides an overview of current and emerging therapeutic strategies targeting mitochondrial dysfunction and its associated pathways in DCM.

### 4.3 Persistent challenges in clinical translation

Despite promising preclinical data, significant translational gaps remain. First, heavy reliance on animal models with distinct metabolic profiles (e.g., acute β-cell toxicity in STZ models vs. insulin resistance in genetic models) limits clinical translatability. Second, Many mitochondria-targeted agents face challenges with bioavailability, tissue-specific delivery, and off-target effects. Clinical trial results have often been less robust than preclinical findings, highlighting species differences and disease complexity. Third, Comorbidities, genetic background, and varying degrees of mitochondrial reserve complicate treatment responses and trial design. Finally, The absence of standardized, non-invasive biomarkers of mitochondrial function hinders early patient stratification, therapeutic monitoring, and demonstration of target engagement in clinical trials.

## 5 Future directions: an integrated roadmap for research and translation

To bridge the gap between mechanistic understanding and clinical impact, future efforts must be guided by an integrated roadmap addressing several key frontiers.

### 5.1 Developing physiologically relevant disease models

Future research must move beyond a one-model-fits-all approach. Model selection should instead be guided by several key principles. First, pathophysiological fidelity is essential, requiring alignment of the model's metabolic phenotype (e.g., insulin deficiency vs. insulin resistance) and the temporal progression of cardiac pathology with the specific human DCM subtype under investigation. Second, comprehensive phenotyping should be implemented, extending beyond glycemic indices to include longitudinal assessments of cardiac function, histopathological evaluation, and detailed mitochondrial functional assays. Third, the incorporation of clinical complexity is critical; priority should be given to aged animals, models with relevant comorbidities (such as hypertension), and human-based systems, including iPSC-derived cardiomyocytes from patients with diabetes, to more accurately recapitulate patient heterogeneity.

### 5.2 Defining the therapeutic window: the concept of reversibility

An important yet unresolved question in diabetic cardiomyopathy concerns the temporal window during which mitochondrial dysfunction remains reversible and therapeutically tractable. Available evidence suggests that mitochondrial abnormalities emerge early in the course of diabetes, often preceding overt structural remodeling or clinical cardiac dysfunction. At these early stages, mitochondrial changes-such as altered substrate utilization, moderate oxidative stress, and impaired mitochondrial dynamics-appear largely functional and potentially reversible (38, 103), as demonstrated by the responsiveness to metabolic interventions, antioxidant therapies, and lifestyle modification in experimental models.

As diabetes progresses, persistent metabolic stress leads to cumulative mitochondrial damage, including sustained oxidative injury, defective mitophagy, mitochondrial DNA damage, and progressive loss of cardiomyocytes through apoptosis and ferroptosis (10, 53). Once these alterations culminate in extensive myocardial fibrosis, irreversible cardiomyocyte loss, and chronic inflammatory remodeling, mitochondrial dysfunction may become self-perpetuating and less amenable to therapeutic reversal. Thus, rather than a discrete irreversible stage, mitochondrial dysfunction in DCM likely transitions along a continuum from functional plasticity to structural fixation. This concept underscores the existence of a critical therapeutic window early in disease progression, during which targeting mitochondrial dysfunction may yield maximal benefit.

### 5.3 Leveraging emerging technologies for target discovery

High-resolution omics technologies are essential to deconvolve DCM's complexity.

The emerging high-throughput omics technologies are beginning to provide more detailed insights into the cellular and spatial heterogeneity underlying diabetic cardiomyopathy. Single-cell RNA sequencing (scRNA-seq) can parse cell type-specific responses and link mitochondrial dysfunction with cell fate transitions (104). A single-cell RNA sequencing study of the hearts of diabetic mice not only confirmed the heterogeneity of cardiomyocytes but also identified a unique fibroblast subpopulation (fibroblasts with high Cthrc1 expression), which highly expresses pro-fibrotic genes and is preferentially expanded in diabetes and spatially associated with the damaged areas (104). This indicates that single-cell RNA sequencing has the ability to distinguish the cellular factors causing dilated cardiomyopathy and to link specific cell states with pathological remodeling. This study demonstrates the ability of single-cell RNA sequencing to address the masked pathogenic cell populations and signaling networks in large-scale transcriptome analysis, thereby providing mechanistic insights into disease progression at the single-cell resolution. Compared with single-cell RNA sequencing technology, the application of spatial omics technologies in diabetic cardiomyopathy is still relatively limited. However, the latest advancements in cardiovascular research indicate that spatial transcriptomics and related spatial omics methods are feasible in depicting regional-specific metabolic remodeling, inflammatory activation, and mitochondrial stress within cardiac tissue structures (105). These technologies can combine molecular characteristics with tissue pathological backgrounds, which is particularly important for this disease (characterized by heterogeneous remodeling of myocardial structure and fibrosis). Although more specific studies are needed for diabetic cardiomyopathy, spatial omics has great potential in identifying local mitochondrial dysfunction and potential microenvironmental sites as precise treatment targets for diabetic cardiomyopathy. The fusion of single-cell data and spatial data will be crucial for constructing a high-fidelity, spatially resolved cell map of the diabetic heart. This map will go beyond correlational observations and establish causal relationships within the tissue background, thereby providing key information for the development of precise intervention measures targeting specific myocardial regions or the most vulnerable cell populations.

### 5.4 Biomarker development for precision medicine

The development of non-invasive, mitochondria-specific biomarkers is paramount for early detection, risk stratification, and therapy monitoring.

Mitochondrial DNA (mtDNA) in the circulation, released into the blood due to mitochondrial damage and oxidative stress, has emerged as a promising biomarker. mtDNA in the plasma of patients with diabetes and cardiovascular metabolic diseases is more prone to oxidative damage and is associated with the severity of cardiovascular complications, serving as a direct indicator of mitochondrial damage (106). In dilated cardiomyopathy, the metabolic transformation of the myocardium is reflected in circulating metabolites. Acylcarnitines as intermediate products of incomplete fatty acid β-oxidation, specific long-chain acylcarnitines (such as C16) accumulate systemically in insulin resistance and diabetes patients. The increase in their plasma levels strongly reflects the low mitochondrial metabolic efficiency and lipotoxicity in the heart (107).

The impaired mitochondrial catabolic metabolism of Branched-chain amino acids (BCAA) is a characteristic of insulin resistance. In diabetes patients, the plasma levels of BCAA and its derivatives (such as α-keto isopropyl acid) are elevated and are associated with diastolic dysfunction and future risk of heart failure, linking systemic mitochondrial substrate processing to cardiac pathology (108).

Mitochondrial-derived peptides (MDPs) are a new type of bioactive micro-peptides encoded by mitochondrial DNA. Examples include humanin and MOTS-c, which are regarded as potential biomarkers and protective factors. These peptides possess properties such as cell protection, metabolic regulation, and anti-apoptosis. In the context of diabetes, the levels of humanin in the circulation change, and a decrease in these levels is associated with cardiovascular complications, suggesting that they may reflect the endogenous mitochondrial stress response capacity (109).

To enable clinical translation, future efforts must move beyond reliance on a single biomarker. Priority should be given to three key directions. First, standardizing detection methodologies for these emerging analytes across different analytical platforms. Second, validating their diagnostic and prognostic performance in large, longitudinal cohorts of patients with DCM, with direct comparison to established imaging-based gold standards. Third, developing integrated biomarker panels that combine mitochondrial indicators (e.g., circulating cell-free mitochondrial DNA and acylcarnitines) with conventional cardiac biomarkers such as NT-proBNP to improve the specificity and sensitivity of early DCM detection. Ultimately, the goal is to establish a mitochondria-informed biomarker signature measurable in readily accessible biological fluids, capable of identifying subclinical myocardial deterioration and enabling timely preventive intervention.

The path to clinical adoption requires technical standardization, validation in large longitudinal cohorts, and the development of integrated biomarker panels combined with conventional markers to achieve the necessary specificity for early DCM.

## 6 Limitations

While this review synthesizes the multifaceted role of mitochondrial dysfunction in DCM and the corresponding therapeutic landscape, the field must overcome several persistent barriers to translate mechanistic insights into clinical benefit. Key limitations include the fragmented study of interconnected pathological pathways, a translational gap exacerbated by inadequate disease models, significant hurdles in targeted delivery of interventions to cardiac mitochondria, and a dearth of validated clinical biomarkers for early diagnosis and monitoring. Addressing these limitations is imperative for translating the promising mechanistic insights outlined here into effective clinical therapies for DCM.

Future research should prioritize the development of clinically relevant disease models, leverage integrated multi-omics approaches to dissect complex pathogenic networks, and foster interdisciplinary collaboration to overcome current barriers in therapeutic delivery and biomarker development, thereby advancing the field toward precision diagnosis and treatment.

## 7 Conclusion

As a complex metabolic heart disease, DCM is closely linked to mitochondrial dysfunction. Beyond its central role in myocardial energy metabolism, mitochondria are key regulators of oxidative stress, metabolic flexibility, and cell fate decisions. Mitochondrial imbalance orchestrates a pathological network characterized by energy metabolic disturbances, oxidative damage, inflammatory activation, and programmed cell death. A systematic understanding of these intertwined mechanisms is crucial for uncovering the pathophysiology of DCM and optimizing therapeutic strategies. Future treatments should focus on mitochondrial targets and develop multi-targeted interventions aimed at simultaneously improving metabolism, antioxidant defenses, and cell survival. The integration of emerging technologies such as single-cell omics and spatial omics may further facilitate the discovery of precision therapeutic approaches, enhancing early warning systems and improving clinical outcomes for patients with DCM.
